# Cellular mechanisms of bone resorption in breast carcinoma

**DOI:** 10.1054/bjoc.2001.1856

**Published:** 2001-07

**Authors:** N C A Hunt, Y Fujikawa, A Sabokbar, I Itonaga, A Harris, N A Athanasou

**Affiliations:** Nuffield Department of Pathology and Bacteriology, University of Oxford, John Radcliffe Hospital, Oxford, Headington OX3 9DU, UK; Department of Pathology, Nuffield Department of Orthopaedic Surgery, Nuffield Orthopaedic Centre, University of Oxford, Oxford, OX3 7LD, UK; Imperial Cancer Research Fund Molecular Oncology Laboratory, University of Oxford, Institute of Molecular Medicine, John Radcliffe Hospital, Oxford, OX3 9DU, UK

**Keywords:** metastasis, breast cancer, osteoclast, bone resorption

## Abstract

The cellular mechanisms that account for the increase in osteoclast numbers and bone resorption in skeletal breast cancer metastasis are unclear. Osteoclasts are marrow-derived cells which form by fusion of mononuclear phagocyte precursors that circulate in the monocyte fraction. In this study we have determined whether circulating osteoclast precursors are increased in number or have an increased sensitivity to humoral factors for osteoclastogenesis in breast cancer patients with skeletal metastases (± hypercalcaemia) compared to patients with primary breast cancer and age-matched normal controls. Monocytes were isolated and cocultured with UMR 106 osteoblastic cells in the presence of 1,25 dihydroxyvitamin D_3_[1,25(OH)_2_D_3_] and human macrophage colony stimulating factor (M-CSF) on coverslips and dentine slices. Limiting dilution experiments showed that there was no increase in the number of circulating osteoclast precursors in breast cancer patients with skeletal metastases (± hypercalcaemia) compared to controls. Osteoclast precursors in these patients also did not exhibit increased sensitivity to 1,25(OH)_2_D_3_ or M-CSF in terms of osteoclast formation. The addition of parathyroid hormone-related protein and interleukin-6 did not increase osteoclast formation. The addition of the supernatant of cultured breast cancer cell lines (MCF-7 and MDA-MB-435), however, significantly increased monocyte-osteoclast formation in a dose-dependent fashion. These results indicate that the increase in osteoclast formation in breast cancer is not due to an increase in the number/nature of circulating osteoclast precursors. They also suggest that tumour cells promote osteoclast formation in the bone microenvironment by secreting soluble osteoclastogenic factor(s). © 2001 Cancer Research Campaign http://www.bjcancer.com


*British Journal of Cancer* (2001) **85**(1), 78–84
© 2001 Cancer Research Campaign
Doi: 10.1054/ bjoc.2001.1856, available online at http://www.idealibrary.com on http://www.bjcancer.com

### Cellular mechanisms of bone resorption in breast carcinoma

**NCA Hunt[1], Y Fujikawa[2], A Sabokbar[2], I Itonaga[2], A Harris[3] and NA Athanasou[2]**

[1]University of Oxford, Nuffield Department of Pathology and Bacteriology, John Radcliffe Hospital, Headington, Oxford OX3 9DU, UK;
[2]Department of Pathology, Nuffield Department of Orthopaedic Surgery, University of Oxford, Nuffield Orthopaedic Centre, Oxford OX3 7LD, UK;
[3]Imperial Cancer Research Fund Molecular Oncology Laboratory, University of Oxford, Institute of Molecular Medicine, John Radcliffe Hospital, Oxford OX3 9DU, UK

**Summary** The cellular mechanisms that account for the increase in osteoclast numbers and bone resorption in skeletal breast cancer metastasis are unclear. Osteoclasts are marrow-derived cells which form by fusion of mononuclear phagocyte precursors that circulate in the monocyte fraction. In this study we have determined whether circulating osteoclast precursors are increased in number or have an increased sensitivity to humoral factors for osteoclastogenesis in breast cancer patients with skeletal metastases (± hypercalcaemia) compared to patients with primary breast cancer and age-matched normal controls. Monocytes were isolated and cocultured with UMR 106 osteoblastic cells in the presence of 1,25 dihydroxyvitamin $D_3$ [1,25$(OH)_2D_3$] and human macrophage colony stimulating factor (M-CSF) on coverslips and dentine slices. Limiting dilution experiments showed that there was no increase in the number of circulating osteoclast precursors in breast cancer patients with skeletal metastases (± hypercalcaemia) compared to controls. Osteoclast precursors in these patients also did not exhibit increased sensitivity to 1,25$(OH)_2D_3$ or M-CSF in terms of osteoclast formation. The addition of parathyroid hormone-related protein and interleukin-6 did not increase osteoclast formation. The addition of the supernatant of cultured breast cancer cell lines (MCF-7 and MDA-MB-435), however, significantly increased monocyte-osteoclast formation in a dose-dependent fashion. These results indicate that the increase in osteoclast formation in breast cancer is not due to an increase in the number/nature of circulating osteoclast precursors. They also suggest that tumour cells promote osteoclast formation in the bone microenvironment by secreting soluble osteoclastogenic factor(s).
© 2001 Cancer Research Campaign http://www.bjcancer.com

**Keywords**: metastasis; breast cancer; osteoclast; bone resorption

Bone destruction is a major complication of advanced malignant disease. It causes bone pain, pathological fracture and hypercalcaemia. Tumour-associated osteolysis and hypercalcaemia is seen in association with haematological malignancies (e.g. myeloma, lymphoma) and solid tumours, mainly carcinomas, where it may occur both in the presence (e.g. cancer of the lung, breast, thyroid, kidney, etc.) and absence (e.g. cancer of the lung, breast, head and neck, kidney, ovary, etc.) of bone metastases (Mundy, 1991; Dodwell, 1992). Bone is one of the commonest sites of metastasis in breast cancer and hypercalcaemia is seen in about one half of patients with clinical evidence of breast carcinoma.

The cellular and molecular mechanisms that account for the bone destruction and consequent hypercalcaemia which occurs in patients with advanced malignant disease are poorly understood. Osteoclasts, multinucleated cells which form part of the mononuclear phagocyte system (Athanasou, 1996), effect the bone resorption in patients with skeletal metastasis (Galasko, 1976; Taube et al, 1994). Osteoclasts are formed by fusion of circulating mononuclear precursors cells of haematopoietic origin. In vitro studies have defined the ontogeny of the osteoclast and characterized the essential cellular and humoral factors which are required for osteoclast differentiation from haematopoietic and circulating osteoclast precursors. In both mouse and man, mononuclear osteoclast precursors circulate in the monocyte fraction and express a monocyte/macrophage rather than an osteoclast phenotype (Udagawa et al, 1990; Quinn et al, 1996; Fujikawa et al, 1996). Osteoclast differentiation from these circulating precursors requires the presence of M-CSF and involves a receptor–ligand interaction with osteoblasts which express a membrane-bound osteoclast differentiation factor (ODF) (Nakagawa et al, 1998; Yasuda et al, 1998).

In previous studies we have shown that tumour-associated macrophages (TAMs) isolated from primary human breast and mouse mammary carcinomas, when cocultured with bone-derived stromal cells in the presence of 1,25$(OH)_2D_3$ and M-CSF, are able to differentiate into multinucleated osteoclasts that are capable of extensive lacunar bone resorption (Quinn et al, 1994, 1998). This finding is of interest with regard to the pathogenesis of tumour osteolysis in breast cancer as, in addition to an increase in osteoclast number, a prominent macrophage infiltrate is commonly found in metastatic breast carcinomas (Bugelski et al, 1987; Van Ravenswaay Claasen et al, 1992). Moreover, osteoclasts are required for growth of breast cancer metastases in bone and tumour osteolysis involves recruitment of osteoclast precursors and activation of mature osteoclasts (Clohisy et al, 1996a, 1996b; Clohisy and Ramnaraine, 1998).

In this study, our aim has been to analyse the cellular mechanisms of bone resorption in breast cancer. As TAMs in metastases of breast cancer are derived from circulating monocytes (Mantovani et al, 1992), we have sought to determine whether the

*Received 20 November 2000*
*Revised 21 March 2001*
*Accepted 27 March 2001*

*Correspondence to:* NA Athanasou number of circulating osteoclast precursors in the monocyte fraction is increased in patients with metastatic breast cancer compared to patients with primary breast cancer alone and normal age-matched females. We have similarly assessed the number of osteoclast precursors in 2 cases of hypercalcaemia of malignancy associated with breast cancer, one with skeletal metastases, the other a case of (non-metastatic) humoral hypercalcaemia of malignancy. We have also analysed the sensitivity of circulating osteoclast precursors in breast cancer patients to various humoral factors required for osteoclastogenesis and determined whether breast carcinoma cells produce soluble factors that promote macrophage–osteoclast differentiation and malignant bone resorption.

### MATERIALS AND METHODS

### Media and sera

Incubations were performed in alpha minimal essential medium (MEM) (Gibco, Paisley, UK) supplemented with glutamine (2 mM), benzyl penicillin (100 IU $ml^{-1}$), streptomycin (100 mg $ml^{-1}$), and 10% fetal calf serum (FCS) (TechGen, London, UK). MEM alone or Hank's balanced salt solution (HBSS) (Gibco, Paisley, UK) were used for cell isolation. Cloned, hormone responsive, calcitonin receptor-negative, osteoblast-like UMR106 cells (derived from a rat osteosarcoma-derived cell line) and human parathyroid hormone-related protein (PTHrP) were obtained from Prof TJ Martin, Melbourne, Australia (Partridge et al, 1981). $1,25(OH)_2D_3$ (Solvay Duphar, NL) and dexamethasone (Sigma, UK) were dissolved in absolute alcohol and stored at –20˚C. Human M-CSF and human interleukin-6 (IL-6) (R&D Systems Europe, Abingdon, UK) were dissolved in MEM/FCS and stored at –20˚C.

### Preparation of monocyte-UMR cocultures on dentine slices and coverslips

Peripheral blood was drawn from 22 patients with breast cancer (± evidence of metastatic disease) (Table 1) and 20 age-matched normal female volunteers. One patient had hypercalcaemia without evidence of metastases. At the time blood was taken, patients were not under chemotherapy or hormone treatment. All patients had normal white cell counts (4–11 × $10^9$ $l^{-1}$), and the monocyte fraction was within the normal range (0.2–0.8 × $10^9$ $l^{-1}$). The blood was collected and diluted 1:1 in MEM, layered over Ficoll-Hypaque (Pharmacia, UK), then centrifuged (693 ***g***), washed and resuspended in MEM/FCS. The number of cells in the resulting suspension of peripheral blood mononuclear cells (PBMCs) was counted in a haemocytometer after lysis of red cells using a 5% (v/v) acetic acid solution.

Dentine slices (4 mm diameter), prepared as previously described (Quinn et al, 1994), and glass coverslips (6 mm diameter) were placed in 96-well tissue culture plates. 2 × $10^4$ UMR106 osteoblast-like cells were added to each well and then cultured on the dentine slices and coverslips for 24 hours in MEM/FCS. The cell suspension of PBMCs (1 × $10^5$ cells $well^{-1}$) was then settled on these coverslips and dentine slices for 2 h. The coverslips and dentine slices were then removed from the wells, washed vigorously in MEM/FCS to remove non-adherent cells, then placed in 24 well tissue culture plates containing 1 ml MEM/FCS. The cell cultures were incubated in the presence of $1,25(OH)_2D_3$ ($10^{-7}$ M), dexamethasone ($10^{-8}$ M) and M-CSF (25 ng $ml^{-1}$) for up to 21 days.

**Table 1** Clinical details of breast cancer patients from whom peripheral blood mononuclear cells were obtained

| | Sex | Age | Bone metastases | Hypercalcaemia |
|---|---|---|---|---|
| Patient 1 | F | 50 | – | – |
| Patient 2 | F | 61 | – | – |
| Patient 3 | F | 46 | + | + |
| Patient 4 | F | 61 | – | + |
| Patient 5 | F | 68 | – | – |
| Patient 6 | F | 52 | – | – |
| Patient 7 | F | 49 | + | – |
| Patient 8 | F | 74 | + | – |
| Patient 9 | F | 60 | – | – |
| Patient 10 | F | 45 | – | – |
| Patient 11 | F | 71 | – | – |
| Patient 12 | F | 64 | – | – |
| Patient 13 | F | 81 | + | – |
| Patient 14 | F | 76 | + | – |
| Patient 15 | F | 69 | + | – |
| Patient 16 | F | 78 | + | – |
| Patient 17 | F | 57 | + | – |
| Patient 18 | F | 77 | + | – |
| Patient 19 | F | 64 | + | – |
| Patient 20 | F | 62 | – | – |
| Patient 21 | F | 62 | + | – |
| Patient 22 | F | 72 | – | – |

### Histochemical and immunohistochemical characterization of cultured cells

Histochemical staining for tartrate-resistant acid phosphatase (TRAP) was carried out using a commercially available kit (Sigma, UK). Cell preparations were fixed in citrate/acetone solution and stained for acid phosphatase, using naphthol AS-BI phosphate as a substrate, in the presence of 1.0 M tartrate; the product was reacted with fast garnet GBC salt (Andersson et al, 1992).

Cell preparations on coverslips were also stained immunohistochemically by an indirect immunoperoxidase technique (Gatter et al, 1984) with the monoclonal antibody 23C6 (a gift of Professor MA Horton, London, UK): this is directed against CD51, the vitronectin receptor (VNR), a highly osteoclast-associated antigen (Horton et al, 1985). Cell preparations were similarly stained with the monoclonal antibody GRS1, directed against CD14 (Schlossman et al, 1995), a macrophage-associated antigen which is known not to be expressed by osteoclasts (Athanasou and Quinn, 1990). To quantify the number of VNR multinucleated cells formed in cocultures on coverslips, the number of these cells were counted in 4 fields of view (10 × objective) on coverslips and the mean taken. Cells containing 2 or more nuclei were considered multinucleated.

### Functional evidence of osteoclast differentiation: detection of lacunar resorption

Functional evidence of osteoclast differentiation was determined by a lacunar resorption assay system using cell culture on dentine slices; the latter provides a smooth-surfaced mineralized substrate for the assessment of lacunar resorption (Boyde et al, 1984). At the end of the coculture period, dentine slices were placed in $NH_4OH$ (1 M) for 30 minutes and cleaned by ultrasonication to remove adherent cells. The slices were then washed with distilled water and stained with 0.5% (v/v) toluidine blue for 3 minutes, then washed again. The extent of lacunar resorption was quantified by counting the number of resorption pits on the slice by light microscopy.

© 2001 Cancer Research Campaign

### Assessment of the number of circulating cells in breast cancer patients and controls

To determine the number of circulating osteoclast precursors in the monocyte fraction of whole blood in breast cancer patients and controls, serial dilutions of PBMCs ($1 \times 10^5$–$1 \times 10^2$ cells) were added to each well and the cocultures maintained in the presence of 1,25(OH)$_2$D$_3$, dexamethasone and M-CSF for up to 21 days. Osteoclast formation in these cocultures was assessed by TRAP and lacunar resorption.

### The effect of osteoclastogenic factors (M-CSF, 1,25(OH)$_2$D$_3$, IL-6 and PTHrP) on osteoclast formation in breast cancer patients and normal controls

In order to determine if osteoclast precursors in the peripheral blood of breast cancer patients relative to normal controls, were more sensitive to the effect on osteoclastogenesis of M-CSF and 1,25(OH)$_2$D$_3$, the extent of osteoclast formation and lacunar resorption was assessed in 21 day UMR 106-monocyte cocultures in the presence of varying concentrations of human M-CSF (1–25 ng ml$^{-1}$) and 1,25(OH)$_2$D$_3$ ($10^{-8}$–$10^{-10}$ M). The addition of IL-6 (10–100 ng ml$^{-1}$) and human PTHrP (10–100 ng ml$^{-1}$), factors which are known to stimulate osteoclast formation and bone resorption in malignancy (Mundy, 1991; Dodwell, 1992; Rosol and Capen, 1992) to monocyte-UMR-106 cocultures was similarly assessed in terms of stimulation of osteoclast formation and lacunar resorption.

### The effect of breast carcinoma cells on osteoclast formation and lacunar resorption

Human breast carcinoma-derived cell lines MDA-MB 435 (Price et al, 1990) and MCF-7 (Soule et al, 1973) were subcultured and then grown for 72 h in MEM/FCS. The supernatant from these cultures was decanted and centrifuged at 693 ***g*** for 7 min prior to filtering in order to remove cellular material and to provide conditioned medium for the subsequent experiments. The conditioned medium was added separately to the monocyte-UMR106 cocultures at final concentrations of 0.01 and 0.1% (v/v). Incubations on glass coverslips were performed over 14 days and those on dentine slices over 21 days.

### Statistical analysis

Each series of experiments was repeated at least 3 times. The results obtained from the experiments were expressed as the percentages (mean ± SEM) of lacunar resorption in experimental cultures to those in each control culture. Significant differences were determined using Student's *t*-test.

### RESULTS

### Histochemical, immunohistochemical and functional characterization of isolated and cultured PBMCs from breast cancer patients

Adherent mononuclear cells isolated from the peripheral blood of breast cancer patients and controls, which had been settled on glass coverslips and cultured for 24 h, were characterized as monocytes on the basis that they expressed CD14, a macrophage-associated antigen which is known not to be expressed by osteoclasts (Athanasou and Quinn 1990), and were entirely negative for the osteoclast markers, TRAP and VNR (Horton et al, 1985). In addition, 24 h culture of PBMCs on dentine slices did not show evidence of lacunar resorption.

### Osteoclast formation and lacunar resorption in monocyte-UMR106 cocultures

Monocytes from all cancer patients and controls, when cocultured with UMR 106 cells, in the presence of 1,25(OH)$_2$D$_3$, dexamethasone and M-CSF, were capable of osteoclast differentiation as shown by the formation of TRAP- and VNR-positive multinucleated cells and lacunar resorption pit formation. After 14 days incubation on glass coverslips, the cocultures contained numerous TRAP (Figure 1) and VNR-positive mononuclear and multinucleated cells. There was no obvious increase in the number of TRAP- and VNR-positive multinucleated cells formed from monocytes derived from controls and breast cancer patients. The mean number (± SEM) of VNR-positive multinucleated cells formed under these conditions was 5.88 (± 1.1) and 6.85 (± 2.40) for controls and breast cancer patients respectively. In 21 day cocultures there was extensive lacunar resorption pit formation on

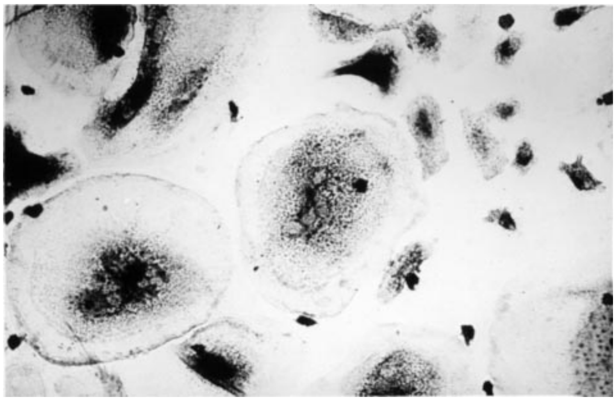

**Figure 1** Numerous tartrate-resistant acid phosphatase-positive multinucleated osteoclasts in a 14 day coculture of UMR-106 osteoblast-like cells and monocytes from breast cancer patients (×250 original magnification)

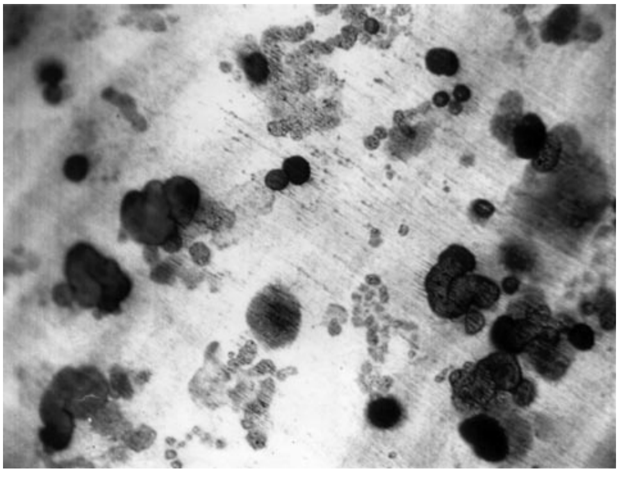

**Figure 2** Extensive lacunar resorption pit formation on dentine slices after 21 days coculture of monocyte-derived osteoclasts and UMR-106 cells (Toluidine blue staining. ×100 original magnification)

 *© 2001 Cancer Research Campaign*

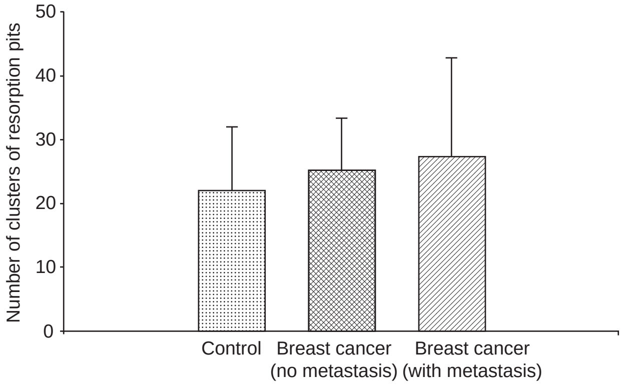

**Figure 3** Lacunar resorption pit formation in cocultures of monocyte-UMR 106 breast cancer patients and age-matched controls dentine slices (Figure 2). Cocultures of PBMCs ($1 \times 10^5$ cells per well) and UMR 106 cells incubated for 21 days showed no increase in lacunar resorption in control and breast cancer (± bone metastases) patients (Figure 3). Neither the number of VNR-positive multinucleated cells nor the extent of lacunar resorption pit formation was associated with the menopausal status of control or cancer patients.

### Comparison of the number of circulating osteoclast precursors in controls and breast cancer patients

Serial dilution of the leucocyte suspension, both from breast cancer patients and normal controls, added to dentine slices, showed that lacunar resorption pits were formed when as few as 100 cells were added to each well. Cocultures of UMR 106 cells with monocytes derived from all breast cancer patients, incubated for 21 days with UMR 106 cells in the presence of $1,25(OH)_2D_3$, M-CSF, and dexamethasone, showed a small but not significant increase in the number of resorption pits formed on dentine slices compared with cell cultures from age-matched female controls (Figure 4).

### Comparison of the effect of osteoclastogenic factors on monocyte–osteoclast formation in controls and breast cancer patients

There was no difference in the sensitivity of monocytes derived from breast cancer patients (± evidence of metastases) compared

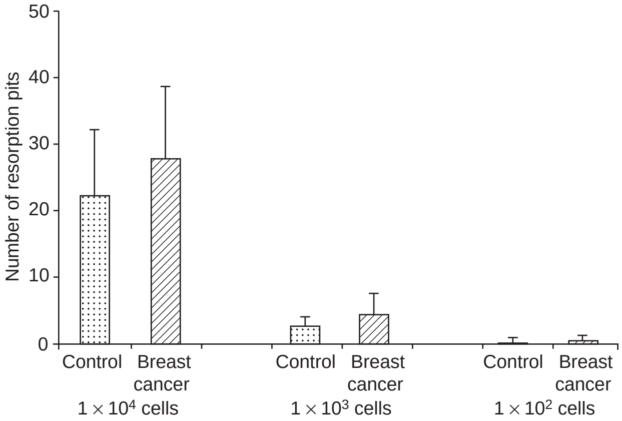

**Figure 4** The effect on resorption pit formation of adding serial dilutions of PBMCs derived from breast cancer patients and age-matched controls to that of monocytes derived from age-matched controls to form osteoclasts in the presence of varying concentrations of $1,25(OH)_2D_3$, M-CSF, IL-6 and PTHrP. In 21 day cocultures of UMR106 cells and monocytes, derived from both breast cancer patients (± evidence of metastases) and controls, incubated in the presence of $10^{-8}$ M and $10^{-9}$ M $1,25(OH)_2D_3$, (with 25 ng ml$^{-1}$ M-CSF and $10^{-8}$ M dexamethasone), no statistically significant difference was seen in the extent of lacunar resorption pit formation on dentine slices (Figure 5). In the presence of $10^{-10}$ M $1,25(OH)_2D_3$, resorption pits were not formed in either breast cancer (± metastases) or control patients. In the presence of 5 ng ml$^{-1}$ M-CSF, there was no evidence of pit formation in cocultures of UMR 106 cells and monocytes derived from either breast cancer or control patient. The lowest concentration of M-CSF at which pit formation was seen in both breast cancer and control patients was 10 ng ml$^{-1}$. No evidence of TRAP expression or lacunar resorption was seen when $1,25(OH)_2$ $D_3$ was replaced with IL-6 or PTHrP. Control and breast cancer patients also showed no difference in resorption when PTHrP or IL-6 was added to the monocyte-UMR 106 cocultures.

### The effect of conditioned medium from cultured breast cancer cells on monocyte–osteoclast differentiation

An increase in TRAP and VNR expression and lacunar resorption was seen in cocultures of UMR106 cells and monocytes incubated in the presence of conditioned medium from cultures of MCF-7 cells or MDA-MB-435 cells compared to control monocyte-UMR106

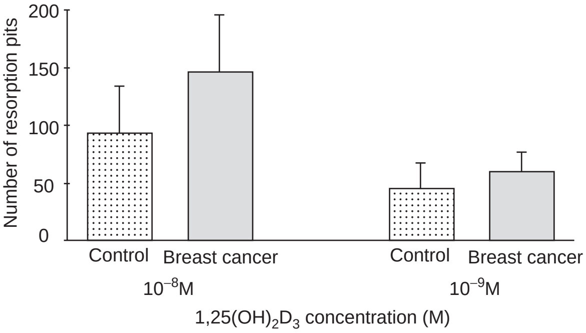

**Figure 5** Lacunar resorption pit formation of UMR 106-monocyte cocultures in breast cancer patients compared to normal controls following the addition of $10^{-8}$ M and $10^{-9}$ M $1,25(OH)_2D_3$

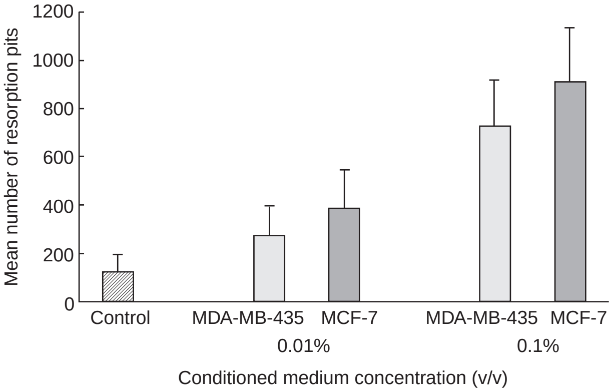

**Figure 6** Dose-dependent increase in lacunar resorption pit formation following the addition of the conditioned medium of cultured MDA-MB 435 and MCF-7 breast cancer cell lines to 21 day monocyte-UMR 106 cocultures

*© 2001 Cancer Research Campaign*

cocultures. The mean number (± SEM) of VNR-positive multinucleated cells formed when conditioned medium from MCF-7 cells and MDA-MB-435 cells was added compared to that of control monocyte-UMR106 cocultures was 7.20 ± 1.29, 8.00 ± 3.31 and 4 ± 0.87 respectively. There was also a marked increase in the extent of lacunar resorption in cocultures on dentine slices under these conditions. The increase in osteoclast formation and resorption pit formation was more marked in cocultures treated with culture supernatant derived from oestrogen receptor-positive MCF-7 cells than in those treated with culture supernatant from oestrogen receptor-negative MDA-MB-435 cells. The addition of culture supernatant from both tumour cell types, however, was found to stimulate osteoclast formation and lacunar resorption in a dose-dependent manner (Figure 6).

### DISCUSSION

Mature osteoclasts, which are the only cells capable of lacunar bone resorption, develop from a marrow-derived population of mononuclear precursor cells which circulate in the monocyte fraction of peripheral blood. M-CSF is an essential humoral requirement for osteoclast formation and $1,25(OH)_2D_3$ is known to promote human osteoclast formation from marrow and circulating precursors in vitro (Tanaka et al, 1993; Fujikawa et al, 1996). Increased osteoclast formation is likely to play a central role in the osteolysis associated with the hypercalcaemia of malignancy and bone metastases in breast cancer. PTHrP production by tumour cells produces hyperparathyroidism-like changes in bone with an increase in osteoclast numbers and lacunar resorptive activity (Mundy, 1991; Dodwell, 1992; Rosol and Capen, 1992). Osteoclasts are also numerous in the vicinity of enlarging breast cancer metastases in bone (Galasko, 1976; Taube et al, 1994).

In this study, we have shown that circulating osteoclast precursors in breast cancer patients, including cases with skeletal metastases and 2 cases of malignant hypercalcaemia, are not increased in number compared to normal controls. We have also shown that these precursors are not more sensitive to the effect of humoral factors ($1,25(OH)_2D_3$, M-CSF, PTHrP and IL-6) which are known to promote osteoclast formation and bone resorption. These results suggest that the increase in osteoclast formation and bone resorption associated with metastatic bone lesions and the hypercalcaemia of malignancy in breast cancer is not due to an increase in the number of circulating mononuclear phagocyte osteoclast precursors or the response of these cells to humoral factors which promote osteoclastogenesis. The addition of conditioned medium from cultured breast cancer cells, however, was found to promote monocyte–osteoclast differentiation, indicating that carcinoma cells in metastatic bone lesions may release soluble factor(s) which promote malignant bone resorption.

A role for increased osteoclast formation in malignant bone resorption and the pathogenesis of the hypercalcaemia of malignancy is indicated by recent studies which have shown that osteoclastogenesis inhibitory factor (osteoprotegerin), a novel secretory factor which blocks the interaction between ODF (RANKL)-expressing bone stromal cells and RANK-expressing osteoclast precursors, induces hypocalcaemia in hypercalcaemic nude mice carrying tumours which cause the hypercalcaemia of malignancy (Akatsu et al, 1998). Although we were able to study only 2 cases of hypercalcaemia of malignancy associated with breast cancer (one of which also had metastatic lesions in bone), we did not find that this condition was associated with an increase in the number of circulating osteoclast precursors found in the monocyte fraction. These precursors were also not more sensitive to the effect of $1,25(OH)_2D_3$ which is essential for osteoclast formation in this in vitro system. Increased osteoclast formation was also not seen after the addition of IL-6 or PTHrP, factors which are known to be released in the humoral hypercalcaemia of malignancy (Rosol and Capen, 1992). The inability of $1,25(OH)_2D_3$ to increase osteoclast formation from circulating precursors may also be of relevance to those cases of hypercalcaemia associated with haematological malignancies in which there is an increase in serum $1,25(OH)_2D_3$ (Suda et al, 1986; Mundy, 1991; Dodwell, 1992; Rosol and Capen, 1992).

An increase in osteoclast numbers and activity is seen in the vicinity of osteolytic bone metastases. A role for increased osteoclast formation in the osteolysis of breast cancer metastases has been suggested by previous studies which have shown that TAMs in primary human breast carcinomas are capable of differentiating into functional bone resorbing osteoclasts when cultured with osteoblast-like cells in the presence of $1,25(OH)_2D_3$ and human M-CSF (Quinn et al, 1998). The results of the present study show that the number of TRAP and VNR-positive cells formed from cells of the monocyte fraction was not increased in breast cancer patients relative to controls. This suggests that the number of primed mononuclear phagocyte osteoclast precursors in the circulation (and thus presumably in the monocyte-derived TAM population), is not increased in breast cancer patients with bone metastases relative to normal controls or breast cancer patients who do not have metastatic disease. It is well-recognized that there is some individual variation in the extent of lacunar resorption by osteoclasts formed from circulating precursors (Fujikawa et al, 1996; Jevon et al, 2000; Shalhoub et al, 2000) and this was evident in both the control and breast cancer patient populations studied; this variation was not related to menopausal status and, given the fact that the number of VNR-positive multinucleated cells formed was not significantly different between the 2 patient groups, most likely reflects differences in the bone-resorbing activity of osteoclasts generated in vitro. Osteoclast precursor cells in the monocyte fraction of breast cancer patients with bone metastases were also not more sensitive to the effect of M-CSF and $1,25(OH)_2D_3$, factors which are required for osteoclast formation. In addition, PTHrP and IL-6, humoral factors which are known both to be produced by metastatic breast carcinoma cells and to play a role in the increased osteoclast formation associated with the tumour osteolysis (Mundy, 1991; Dodwell, 1992; Rosol and Capen, 1992), did not stimulate osteoclastogenesis.

In this study, we found that the conditioned medium derived from cultures of breast cancer cells stimulated osteoclast formation from circulating precursors. As these mononuclear phagocyte precursors are part of the (monocyte-derived) TAM population of metastatic tumours, these findings are of significance with regard to the localized bone resorption that accompanies skeletal metastasis. Our results are supported by the recent findings of Grano et al (2000) who showed that an increased number of TRAP-positive cells is formed in human bone marrow cultures to which the conditioned medium of cultured breast cancer cells is added; the osteoclastic nature of the multinucleated cells formed in these human marrow cultures, however, was not confirmed functionally (i.e. by demonstration of lacunar resorption). Tumour cells are known to secrete factors some of which (e.g. IL-3 and GM-CSF) have been shown to promote osteoclast formation from monocyte precursors (Fujikawa et al, 2001). Lee et al (1991) showed that cultures of a cell line derived from a murine mammary carcinoma that induced

 © 2001 Cancer Research Campaign hypercalcaemia was associated with the release of an osteoclast CSF. Other cultured breast cancer cell lines, known to produce prostaglandins and leukaemia inhibitory factor, have also been shown to promote osteoclastogenesis via a stromal cell-dependent pathway (Akatsu et al, 1998, Ono et al, 1998).

1,25$(OH)_2D_3$ and M-CSF receptors have been demonstrated on breast cancer cells (Suda et al, 1986; Kacinsky 1998) and it is possible that 1,25$(OH)_2D_3$ and M-CSF enhance osteoclast formation by an effect on tumour cells (possibly through ODF production) as our results suggest that these factors do not appear to act directly on osteoclast precursors. A role for ODF in breast cancer-induced osteolysis is suggested by the observation that breast cancer cells stimulate osteoblast production of ODF (Thomas et al, 1998); breast cancer cells are also known to express RANK, which interacts with ODF, and to produce OPG, the soluble decoy receptor for ODF. Taken together, these findings point to a role for tumour cell-derived factors in controlling osteoclast formation from mononuclear phagocyte precursors.

Our data indicate that the increase in osteoclast formation which occurs in both malignant hypercalcaemia and in metastatic bone lesions in breast cancer is not due to a change in either the number or nature of circulating osteoclast precursors, but is most likely a function of the bone microenvironment. All the cellular and humoral elements which are required for osteoclast differentiation can be found in the microenvironment of a metastatic carcinoma in bone. Our results suggest that tumour cells directly influence monocyte/macrophage–osteoclast differentiation through the release of soluble factors which promote osteoclastogenesis. This leads to an increase in bone resorption and is likely to be one of the means whereby increased osteoclast numbers and bone resorption develop in skeletal metastasis. Further studies are needed to determine whether a similar mechanism underlies the malignant bone resorption which is seen in the (non-metastatic) humoral hypercalcaemia of malignancy.

### ACKNOWLEDGEMENTS

This study was supported by the Oxfordshire Health Authority. The authors thank Mrs C Costar for typing the manuscript.

### REFERENCES

Akatsu T, Ono K, Katayama Y, Tamura T, Nishikawa M, Kugai N, Yamamoto M and Nagata N (1998a) The mouse mammary tumor cell line MMT06562 produces prostaglandin E2 and leukemia inhibitory factor and supports osteoclast formation *in vitro* via a stromal dependent pathway. *J Bone Miner Res* **13**: 400–408

Akatsu T, Murakami T, Ono K, Nishikawa M, Tsuda E, Mochizuki SI, Pujise N, Higashio K, Motoyoshi K, Yamamoto M and Nagata N (1998b) Osteoclastogenesis inhibitory factor exhibit hypocalcemic effect in normal mice and in hypercalcemic nude mice carrying tumors associated with humoral hypercalcemia of malignangy. *Bone* **23**: 495–498

Andersson G, Ek-Rylander B and Minkin C (1992) Acid phosphatases. In: Rifkin BR, Gay CV (eds). *Biology and physiology of the osteoclast*, pp 55–80. Boca Raton: CRC Press

Athanasou NA (1996) The cellular biology of bone-resorbing cells. (Review). *J Bone Joint Surgery (A)* **78**: 1096–1112

Athanasou NA and Quinn J (1990) Immunophenotypic differences between osteoclasts and macrophage polykaryons: immunohistological distinction and implications for osteoclast ontogeny and function. *J Clin Pathol* **43**: 997–1004

Boyde A, Ali NN and Jones SJ (1984) Resorption of dentine by isolated osteoclasts *in vitro*. *Br Dent J* **156**: 216–220

Bugelski PJ, Corwin SP, North SM, Kirsh RL, Nicolson GL and Poste G (1987) Macrophage content of spontaneous metastases at different stages of growth. *Cancer Res* **47**: 4141–4145

Clohisy DR and Ramnaraine ML (1998) Osteoclasts are required for bone tumors to grow and destroy bone. *J Orthop Res* **16**: 660–666

Clohisy DR, Ogilvie CM, Carpenter RJ and Ramnaraine MLR (1996a) Localised tumor-associated osteolysis involves the recruitment and activation of osteoclasts. *J Orthop Res* **14**: 2–6

Clohisy DR, Palkert D, Ramnaraine ML, Pekurovsky I and Oursler MJ (1996b) Human breast cancer induces osteoclast activation and increases the number of osteoclasts at sites of tumor osteolysis. *J Orthop Res* **14**: 396–402

Dodwell DJ (1992) Malignant bone resorption: cellular and biochemical mechanisms. *Annals of Oncology* **3**: 257–267

Fujikawa Y, Quinn JMW, Sabokbar A, Mcgee Jo'd and Athanasou NA (1996) The human mononuclear osteoclast precursor circulates in the monocyte fraction. *Endocrinology* **139**: 4058–4060

Fujikawa Y, Sabokbar A, Neale SD, Itonaga I, Torisu T and Athanasou NA (2001) The effect of macrophage-colony stimulating factor and other humoral factors (Interleuken-1, -3, -6, -11, tumour necrosis factor-α granulocyte macrophage-colony stimulating factor) on human osteoclast formation from circulating cells. Bone (In Press)

Galasko CBS (1976) Mechanisms of bone destruction in the development of skeletal metastasis. *Nature* **276**: 726–728

Gatter KC, Falini B and Mason DY (1984) The use of monoclonal antibodies in histological diagnosis, In: Anthony PP, Mac Sween RNM (eds) Recent advances in histopathology, No 12. Edinburgh: Churchill Livingstone 35–67

Grano M, Morgi G, Minielli V, Cantatore FP, Colucci S and Zambonin Zallone A (2000) Breast cancer cell line MDA-231 stimulates osteoclastogenesis and bone resorption in human osteoclasts. *Bioch Biophys Res Commun* **270**: 1097–1100

Horton MA, Lewis D, Mcnulty K, Pringle JAS and Chambers TJ (1985) Monoclonal antibodies to osteoclastomas (giant cell bone tumours): definition of osteoclast specific cellular antigens. *Cancer Res* **45**: 5663–5669

Jevon M, Hirayama T, Wass J, Brown M, Sabokbar A and Athanasou NA (2000) Osteoclast formation from circulating precursors in primary and secondary osteoporosis. *J Bone Mineral Res* **15**: (Suppl 1): 593

Kacinsky BM (1995) CSF-1 and its receptor in ovarian, endometrial and breast cancer. *Ann Med* **27**: 79–85

Lee MY, Eyre DR and Osborne WRA (1991) Isolation of murine osteoclast colony-stimulating factor. *Proc Natl Acad Sci USA* **88**: 8500–8504

Mantovani A, Botlazzi B, Coloth F, Sozzani S and Ruco L (1992) The origin and function of tumour-associated macrophages. *Immunol Today* **13**: 265–276

Mundy GR (1991) Mechanisms of osteolytic bone destruction. *Bone*. **12**: 51S–56S

Nakagawa N, Kinosaki M, Yamaguchi K, Shima N, Yasuda H and Yano K, et al (1998) RANK is the essential signalling receptor for osteoclast differentiation factor in osteoclastogenesis. *Biochem Biophys Res Commun* **253**: 395–400

Ono K, Akatsu T, Murakami T, Wada S, Nishikawa M, Kugai N, Yamamoto M, Matsuura N and Nagata N (1998) Mouse mammary carcinoma cell line (BALB/c-MC) stimulates osteoclast formation from mouse bone marrow cells through cell-to-cell contact. *Bone* **23**: 27–32

Partridge N, Alcorn D, Michelangeli V, Kemp E, Ryan G and Martin TJ (1981) Functional properties of hormonal responsive cultured normal and malignant rat osteoblastic cells. *Endocrinology* **108**: 213–219

Price JE, Polyzos A, Zhang RD and Daniels LM (1990) Tumorigenicity and metastasis of human breast carcinoma cell lines in nude mice. *Cancer Res* **50**: 717–721

Quinn J, Matsumura Y, Tarin D, Mcgee Jod and Athanasou NA (1994) Cellular and hormonal mechanisms associated with malignant bone resorption. *Lab Invest* **71**: 465–471

Quinn J, Sabokbar A and Athanasou NA (1996) Cells of the mononuclear phagocyte series differentiate into osteoclastic lacunar bone-resorbing cells. *J Pathol* **179**: 106–111

Quinn JMW, Mcgee Jo'd and Athanasou NA (1998) Human tumour-associated macrophages differentiate into osteoclastic bone resorbing cells. *J Pathol* **184**: 31–36

Rosol TJ and Capen CC (1992) Mechanisms of cancer-induced hypercalcaemia. *Lab Invest* **67**: 680–702

Schlossman SF, Boumsell L, Gilks W, Harlan JM, Kishimoto T and Morimoto C, et al (1995) Leucocyte typing V: white cell differentiation antigens. Oxford: Oxford University Press

Shalhoub V, Elliott G, Chiu L, Manoukian M, Kelley M, Hawkins N, Davy E, Shimamoto G, Beck J, Kaufman SA, Van G, Scully S, Qi M, Grisanti M, Dunstan C, Boyle W and Lacey D (2000) Characterization of osteoclast prescursors in human blood. *Br J Haematol* **111**: 501–512

Soule HD, Vazquez J, Long A, Albert S and Brennan M (1973) A human cell line from a pleural effusion derived from a breast carcinoma. *J Natl Cancer Inst* **51**: 1409–1416

© 2001 Cancer Research Campaign 

Suda T, Miyaura C, Abe E and Kuroki T (1986) Modulation of cell differentiation, immune response and tumor promotion by vitamin D components. In; Bone and Mineral Research. Vol 4, Peck W (ed), *Elsevier BV* 1– 47

Tanaka S, Takahashi N, Udagawa N, Tamura T, Akatsu T, Stanly ER, Kurokawa T and Suda T (1993) Macrophage colony stimulating factor is indispensable for both proliferation and differentiation of osteoclast progenitors. *J Clin Invest* **91**: 257–263

Taube T, Elomaa I, Blomqvist C, Beneton MNC and Kanis JA (1994) Histomorphometric evidence for osteoclast-mediated bone resorption in metastatic breast cancer. *Bone* **15**: 161–166

Thomas RJ, Guise TA, Yin JJ, Elliott J, Horwood NJ, Martin TJ and Gillespie MT (1998) Breast cancer cells stimulate osteoblastic osteoclast differentiation factor (ODF) to support osteoclast formation. *Bone* (Suppl) **23**: S378

Udagawa N, Takahashi N, Akatsu T, Sasaki T, Nishihara T, Kogan T, Martin TJ and Suda T (1990) Origin of osteoclasts: mature monocytes and macrophages are capable of differentiating into osteoclasts under a suitable microenvironment prepared by bone-marrow derived stromal cells. *Proc Natl Acad Sci USA* **87**: 7260–7264

Van Ravenswaay Claasen HH, Kluin PM and Fleuren G (1992) Tumor infiltrating cells in human cancer: on the possible role of CD16+ macrophages in antitumor cytotoxicity. *Lab Invest* **67**: 166

Yasuda H, Shima N, Nakagawa N, Yamaguchi K, Kinosaki M and Mochizuki S-I, et al (1998) Osteoclast differentiation factor is a ligand for osteoprotegerin/osteoclastogenesis-inhibitory factor and identical to TRANCE/RANKL. *Proc Natl Acad Sci USA* **95**: 3597–3602

*© 2001 Cancer Research Campaign*
